# Intravitreal aflibercept versus bevacizumab for treatment of myopic choroidal neovascularization

**DOI:** 10.1038/s41598-018-32761-z

**Published:** 2018-09-26

**Authors:** Jia-Kang Wang, Tzu-Lun Huang, Pei-Yao Chang, Yen-Ting Chen, Chin-Wei Chang, Fang-Ting Chen, Yung-Ray Hsu, Yun-Ju Chen

**Affiliations:** 1 Department of Ophthalmology, Far Eastern Memorial Hospital, New Taipei City, Taiwan; 2 Department of electrical engineering, Yuan Ze University, Taoyuan City, Taiwan; 3 Department of Medicine, National Yang Ming University, Taipei City, Taiwan; 4 Department of Healthcare Administration and Department of Nursing, Oriental Institute of Technology, New Taipei City, Taiwan; 5 Department of Medicine, National Taiwan University, Taipei City, Taiwan; 6 Department of Medicine, National Cheng Kung University, Tainan City, Taiwan

## Abstract

The authors performed a retrospective and comparative study to compare the efficacy of intravitreal aflibercept and bevacizumab for patients with myopic choroidal neovascularization (mCNV). The patients with treatment-naïve mCNV received 1 + PRN intravitreal bevacizumab from March 2008 to February 2013, while from March 2013 to July 2016 patients were treated by 1 + PRN intravitreal aflibercept, all with monthly follow-up for 12 months. Primary outcome measures included change in central foveal thickness (CFT) in 1 mm by spectral-domain optic coherence tomography, and best corrected visual acuity (BCVA) at month 12. Complications after injections were recorded. The intra-group changes in CFT and BCVA were compared with Wilcoxon signed rank test, the between-group difference compared with Wilcoxon rank sum test. Fisher’s exact test was used for categorical comparison between groups. Seventy-eight eyes of 78 patients were collected. There were 42 eyes in bevacizumab group, with mean age of 53.2 ± 5.4 years and 27 female patients of them. The mean BCVA significantly improved from baseline 0.56 ± 0.35 logMAR to 0.35 ± 0.35 logMAR at Month 12 after bevacizumab treatment (p < 0.001). The mean CFT significantly decreased from baseline 315.3 ± 25.6 μm to 253.7 ± 24.4 μm at Month 12 following intravitreal bevacizumab (p < 0.001). There were 36 eyes in aflibercept group, with mean age of 52.8 ± 6.8 years and 24 female patients of them. The mean BCVA significantly improved from baseline 0.61 ± 0.47 logMAR to 0.38 ± 0.41 logMAR at Month 12 after aflibercept treatment (p < 0.001). The mean CFT significantly decreased from baseline 328.2 ± 19.8 μm to 241.8 ± 27.2 μm at Month 12 following intravitreal aflibercept (p < 0.001). The baseline demographics, lens status, axial length, refractive errors, duration of symptoms, BCVA, and CFT did not differ significantly between groups (p > 0.05). There was no significant difference between bevacizumab and aflibercept groups in BCVA and CFT from Month 1 to Month 12 (p > 0.05). Injection number of aflibercept was 2.11 ± 0.41, less than that of bevacizumab (3.23 ± 0.38) during 12-month period (p = 0.01). There were no systemic thromboembolic event, elevated intraocular pressure, retinal detachment, or infectious endophthalmitis following injections in both groups. We concluded that both aflibercept and bevacizumab can effectively treat choroidal neovascularization in high myopes. Intravitreal aflibercept had similar efficacy but less treatment number than bevacizumab for mCNV during 12-month period.

## Introduction

Subfoveal or juxtafoveal myopic choroidal neovascularization (mCNV) is an important cause of visual impairment in highly myopic patients^1,2^. Elevated intraocular level of vascular endothelial growth factor (VEGF) was associated with formation of mCNV^3^. Intravitreal administration of different anti-VEGF agents was proven to be effective for treating mCNV, such as bevacizumab (Avastin™, Genentech Inc., South San Francisco, CA, USA), and aflibercept (Eylea™, Regeneron Pharmaceuticals, Inc., Tarrytown, NY, USA, and Bayer Pharma AG, Berlin, Germany)^4–19^. Bevacizumab and aflibercept showed greater ability to manage mCNV than either verteporfin photodynamic therapy or sham injections in prior randomized control trials^4,5,16^. However, head-to-head comparison of efficacy between bevacizumab and aflibercept for mCNV was lacking. To our knowledge, this was the first study to compare the clinical outcomes of bevacizumab and aflibercept in treating mCNV.

## Methods

The protocol of the study which followed the Declaration of Helsinki was approved by institutional review board of Far Eastern Memorial Hospital in Taiwan. All the patients signed the informed consent to agree receiving intravitreal injections and participating the study. This is a retrospective, comparative, and non-randomized study. Patients with treatment-naïve mCNV were consecutively collected. All the highly myopic patients had age more than 18 years and axial length more than 26 mm. Pseudophakic and phakic patients were allowed for inclusion. They presented with best-corrected visual acuity (BCVA) between 20/400 to 20/40, subfoveal or juxtafoveal choroidal neovascularization with or without intramacular or submacular fluid on spectral domain optical coherence tomography (SD-OCT, RTVue, Optovue Inc., San Francisco, CA, USA) using 6 radial line scans through the fovea, submacular leakage on fundus fluorescein angiography (TRC-NW7SF, Topcon Inc., Tokyo, Japan), with or without accompanying submacular hemorrhage on fundus color photography. We excluded pregnant or nursing women, and also the patients with the history of thromboembolic events, major surgery within the previous 3 months or planned within the next 28 days, uncontrolled hypertension, known coagulation abnormalities or current use of anticoagulative medication other than aspirin, prior macular photocoagulation or photodynamic therapy, prior intraocular surgeries within 3 months, presence of active infectious disease or intraocular inflammation, intraocular pressure more than 25 mmHg, or presence of iris neovascularization/vitreous hemorrhage.

Almost all the patients with treatment-naïve mCNV received bevacizumab treatment in our clinics before March 2013. Aflibercept was available after March 2013, and we mostly changed the first-line therapy to aflibercept for mCNV. Patients treated from March 2008 to February 2013 received intravitreal bevacizumab 1.25 mg in 0.05 mL. From March 2013 to July 2016 patients received intravitreal aflibercept 2 mg in 0.05 mL. Aflibercept or bevacizumab was performed 1 + PRN injection intravitreally with monthly follow-up for at least 12 months. The 1 + PRN regimen included baseline treatment and then retreatment while one or more of the criteria were met as follows: (1) BCVA decrease equal or more than one line from the previous visual examination; (2) central foveal thickness (CFT) increase equal or more than 50 μm from the previous SD-OCT examination; (3) Persistent or recurrent cystic macular changes, submacular fluid, or pigment epithelial detachment on SD-OCT; (4) Persistent or recurrent submacular hemorrhage on the fundus examination. BCVA in Snellen chart (converting into logMAR and EDTRS letters for statistical comparison)^20^, intraocular pressure via pneumotonometer (CT-80, Topcon Inc., Tokyo, Japan), biomicroscope of anterior segment, SD-OCT of macula, and fundus color photography were examined during follow-up visits. The follow-up SD-OCT scans used the baseline scan as a reference. Visual testing was done in the same room at each visit. Primary outcome measures included change in CFT and BCVA at month 12. The BCVA and CFT of the baseline were compared with those from Month 1 to Month 12 using Wilcoxon signed rank test within the bevacizumab and aflibercept groups. The between-group numerical differences were compared with Wilcoxon rank sum test. Fisher’s exact test was used for categorical comparison between groups. P value less than 0.05 was considered significant.

## Results

**Figure 1:**
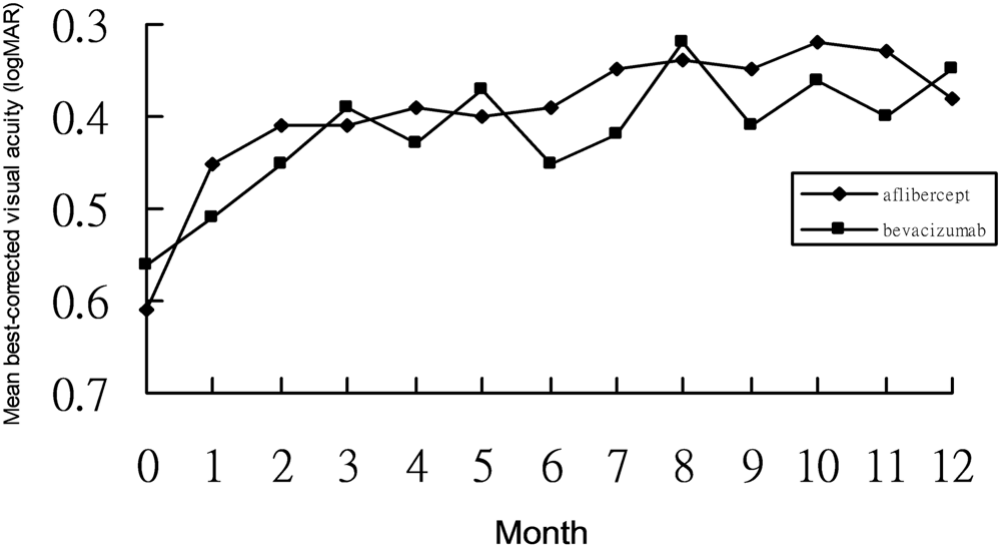
Changes of best-corrected visual acuity from baseline to month 12 in patients with myopic choroidal neovascularization treated by intravitreal aflibercept or bevacizumab.

**Figure 2:**
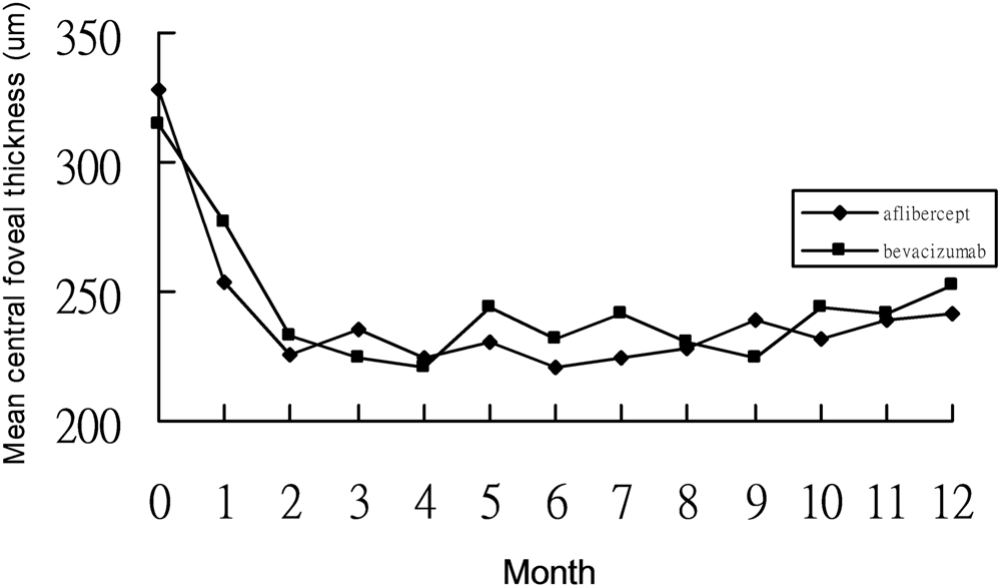
Changes of central foveal thickness from baseline to month 12 in patients with myopic choroidal neovascularization treated by intravitreal aflibercept or bevacizumab.

Intravitreal bevacizumab was performed on 42 eyes with mCNV (Table 1). The mean age was 53.2 ± 5.4 years, and 27 patients of them were female. Of 42 patients, there were 28 phakic ones, whose refractive errors were −7.1 ± 3.1 diopters. The mean axial length was 28.4 ± 3.1 mm. The duration of symptoms was 20.6 ± 7.1 days. There were 2 eyes combined with dome shaped macula, and 3 with myopic foveoschisis among these patients in bevacizumab group. The baseline mean BCVA was 0.56 ± 0.35 logMAR. The mean BCVA significantly improved every month from Month 1 (0.51 ± 0.37 logMAR) to Month 12 (0.35 ± 0.35 logMAR) after bevacizumab treatment (p < 0.05) (Fig. 1). The mean changes from baseline to final BCVA were −0.21 ± 0.33 logMAR (10.5 ± 18.9 letters). There were 61.9% of patients having final BCVA more than or equal to 20/40 (Table 2). More than or equal to 3-line gains were found in 42.8% of the patients after one-year bevacizumab treatment, and one-line loss in 2 of 42 eyes (4.7%). The mean CFT significantly decreased from 277.1 ± 26.8 μm at Month 1 to 253.7 ± 24.4 μm at Month 12 following intravitreal bevacizumab, comparing to the mean baseline CFT as 315.3 ± 25.6 μm (p < 0.05) (Fig. 2). The mean bevacizumab injection number during 12 months was 3.23 ± 0.38. Within the first 3 months, mean 2.71 ± 0.28 bevacizumab injections were required. From Month 6 to 12, 23 of 42 patients (54.7%) did not need any bevacizumab therapy.

**Table 1:** Comparison of baseline data between intravitreal aflibercept and bevacizumab for myopic choroidal neovascularization.

	Aflibercept (n = 36)	Bevacizumab (n = 42)	P value
Age (years)	52.8 ± 6.8	53.2 ± 5.4	0.27
Gender (male: female)	12:24	15:27	0.19
Lens status (phakic: pseudophakic)	25:11	28:14	0.35
Axial length (mm)	29.8 ± 2.7	28.4 ± 3.1	0.11
Refractive errors in phakic eyes (diopter)	−8.2 ± 1.5	−7.1 ± 3.1	0.18
Duration of symptoms (days)	18.1 ± 6.2	20.6 ± 7.1	0.25
Central foveal thickness ( μm)	328.2 ± 19.8	315.3 ± 25.6	0.24
Best corrected visual acuity (logMAR)	0.61 ± 0.47	0.56 ± 0.35	0.18

**Table 2:** Comparison of clinical data after 12-month treatment of intravitreal aflibercept or and bevacizumab for myopic choroidal neovascularization.

	Aflibercept (n = 36)	Bevacizumab (n = 42)	P value
Final BCVA (logMAR)	0.38 ± 0.41	0.35 ± 0.35	0.21
Changes in BCVA (logMAR)	−0.23 ± 0.39	−0.21 ± 0.33	0.44
Changes in BCVA (ETDRS letters)	11.2 ± 15.4	10.5 ± 18.9	0.24
Final BCVA ≥ 20/40	21/36 (58.3%)	26/42 (61.9%)	0.35
BCVA gains ≥ 3 lines	18/36 (50.0%)	19/42 (42.8%)	0.14
BCVA loss ≥ 1 line	0/36 (0%)	2/42 (4.7%)	0.09
Final CFT (μm)	241.8 ± 27.2	253.7 ± 24.4	0.36
Changes in CFT (μm)	−87.5 ± 37.6	−62.4 ± 28.4	0.12
Injection number	2.11 ± 0.41	3.23 ± 0.38	0.01

BCVA: best-corrected visual acuity

CFT: central foveal thickness.

Intravitreal aflibercept was performed on 36 eyes with mCNV (Table 1). The mean age was 52.8 ± 6.8 years, and 24 patients of them were female. Of 36 eyes, there were 25 phakic ones, whose refractive errors were −8.2 ± 1.5 diopters. The mean axial length was 29.8 ± 2.7 mm. The duration of symptoms was 18.1 ± 6.2 days. There were one eye combined with dome shaped macula, and 4 with myopic foveoschisis among these patients in aflibercept group. The baseline mean BCVA was 0.61 ± 0.47 logMAR. The mean BCVA significantly improved every month from Month 1 (0.45 ± 0.38 logMAR) to Month 12 (0.38 ± 0.41 logMAR) after aflibercept treatment (p < 0.05) (Fig. 1). The mean changes from baseline to final BCVA were −0.23 ± 0.39 logMAR (equal to 11.2 ± 15.4 letters). There were 58.3% of patients having final BCVA more than or equal to 20/40 (Table 2). Half of the patients gained more than or equal to 3 lines after one-year aflibercept treatment, and nobody experienced visual loss. The mean CFT significantly decreased from 254.1 ± 22.4 μm at Month 1 to 241.8 ± 27.2 μm at Month 12 following intravitreal aflibercept, comparing to the mean baseline CFT as 328.2 ± 19.8 μm (p < 0.05) (Fig. 2). The mean aflibercept injection number during 12 months was 2.11 ± 0.41. Within the first 3 months, mean 1.98 ± 0.29 aflibercept injections were required. From Month 6 to 12, 28 of 36 patients (77.7%) did not need any aflibercept therapy.

Baseline clinical data including age, gender, lens status, axial length, refractive errors, duration of symptoms, mean BCVA, and mean CFT were comparable between bevacizumab and aflibercept groups (p > 0.05). At all time points from month 2 to month 12, CFT and BCVA were not significantly different between two groups (p > 0.05). There was also no significant difference between two groups in mean visual gains and mean CFT changes (p > 0.05). Aflibercept had significantly lower injection number than bevacizumab during 12 months (p = 0.03).

The injections were well tolerated in all patients. No serious ocular or systemic complications were observed, such as retinal detachment, retinal pigment epithelial tears, infectious endophthalmitis, and thromboembolic events in either aflibercept or ranibizumab groups. The most common side effect was local hyperemia or subconjunctival hemorrhage at the site of injection.

## Discussion

Choroidal neovascularization can cause severe visual impairment in highly myopic patients after long-term follow-up without intervention^1^. Photodynamic therapy with verteporfin demonstrated short-term ability for choroidal neovascularization regression and visual stabilization in high myopes^21^. However, long-term results showed progressive visual deterioration and macular chorioretinal atrophy after photodynamic therapy for mCNV^22,23^.

The pathophysiology of mCNV involves the presence of angiogenic stimulant VEGF. Decreased aqueous VEGF amount and accompanying regressed mCNV were found after intravitreal injection of anti-VEGF agents^24,25^. Bevacizumab is a full-length recombinant humanized monoclonal antibody against the VEGF-A. Ruiz-Moreno and coauthors performed a multicentric and randomized study to compare two treatment groups for mCNV: 1 + PRN photodynamic therapy and 3 + PRN intravitreal bevacizumab^4^. Visual improvement from the baseline was 11.2 letters after one-year follow-up using bevacizumab treatment for mCNV. Increased 10 or more letters of BCVA was found in 52% of patient in bevacizumab group, significantly more than that in 14% of patients in photodynamic therapy group. The mean 3.5 bevacizumab injections were required for management of mCNV during one-year period. Parodi and associates compared the efficacy of 3 randomized groups for juxtafoveal mCNV: laser photocoagulation, photodynamic therapy, and 1 + PRN intravitreal bevacizumab^5^. The authors found bevacizumab injections resulted in visual improvement from mean 0.6 logMAR at baseline to 0.42 logMAR at Year 2, which were better than visual deterioration in photodynamic therapy (mean 0.52 logMAR to 0.72 logMAR) through two-year period. Ruiz-Moreno and colleagues performed a single-armed multicentric study for one year^6^. Visual gains of 8.7 letters were achieved following 1 + PRN intravitreal bevacizumab for 107 eyes with mCNV. Increased 15 or more letters of BCVA was found in 30% of the patients. Single injection was required in 60% of the patients. Kuo and coauthors revealed the mean BCVA improved from baseline 1.09 logMAR to 0.77 logMAR at Month 12 using 1 + PRN bevacizumab monotherapy in a Taiwanese case series of mCNV^7^. Patients only received an average of 2.2 injections. In this study, we had mean 10.5-letter visual gain after one-year 1 + PRN bevacizumab injections, comparable to the results described in prior studies in mean 8.7 to 11.2-letter improvement^4,6^. There were 42.8% of patients having at least 15-letter (3-line) gains after our bevacizumab monotherapy, between 30% to 44% in previous reports^6,8^. We used average 3.23 bevacizumab injections during one-year period, between average 1.8 to 3.5 injections in the preceding reserches^4,6,7^. Most of the intravitreal bevacizumab were administered in the first 3 months, which was compatible to the findings in the previous reports^6,8^. None of our patients reported serious adverse events during ranibizumab monotherapy, similar to the safety reports from previous studies^4–15^.

Intravitreal bevacizumab showed promising long-term visual and anatomical outcomes for treatment of mCNV. Chen and coworkers demonstrated 1 + PRN intravitreal 2.5-mg bevacizumab could cause nearly −0.3 logMAR visual gains at the end of 2-year follow-up in Taiwan^9^. In a prospective case series, Gharbiya and associates showed the mean BCVA gained from baseline 30.1 letters to 45.5 letters at the end of 3-year study period, using treatment of only initial 3 monthly intravitreal bevacizuamb for patients with mCNV^10^. A multicentric study revealed nearly mean 7-letter BCVA increased after either 1 + PRN or 3 + PRN intravitreal bevacizumab for 4 years^11^. Final BCVA remained stable without significant improvement comparing to the baseline BCVA following 5 or 6-year bevacizumab treatment for mCNV^12–14^. In a 7-year follow-up study, mean BCVA improved by approximately 1.8 lines in 17 patients with mCNV receiving intravitreal bevacizumab treatments^15^.

Aflibercept is a decoy receptor fusion protein, consisting of the third domain of VEGF receptor 2 and the second domain of human VEGF receptor 1, which are fused to the Fc domain of human IgG1. Aflibercept was approved as the anti-VEGF drug for mCNV by European Medicines Agency. The MYRROR study was a randomized controlled trial, which compared the efficacy of 1 + PRN intravitreal aflibercept and sham injection treatment for mCNV^16^. During the first 6 months after interventions, aflibercept injections revealed significantly better visual gains (mean 12.1 letters) than sham group (mean 2.0-letters). The visual improvement from the baseline can maintain on 13.5 letters after one-year follow-up using aflibercept treatment for mCNV. Increased 15 or more letters of BCVA was found in 36.9% of the patients. The authors performed the mean 4.2 aflibercept injections to manage mCNV during one-year period, and most of the injections were performed in the first 2 months with minimal subsequent re-injections. Low incidences of ocular (3.3%) and nonocular (4.4%) serious adverse events were reported. Other prior case series also demonstrated similar efficacy of aflibercept to treat choroidal neovascularization in highly myopic eyes^17–19^. Pece and Milani reported mean 10.6-letter visual gains at the end of 1-year follow-up using intravitreal aflibercept for mCNV^19^. Korol and coauthors demonstrated 16% of the eyes had an improvement in BCVA of 3 lines or more using 1 + PRN aflibercept monotherapy in one year^18^. The patients underwent average 2.6 injections within a year. Brue and coauthors also showed mean BCVA improvement from baseline 0.69 logMAR to 0.15 logMAR at 18 months following aflibercept injections in high myopes^17^. In this study, we had mean 11.2-letter visual gain after one-year 1 + PRN aflibercept injections, comparable to the results of prior studies in mean 10.6 to 13.5-letter improvement^16,19^. There were 50.0% of patients having at least 15-letter increase following our aflibercept monotherapy, equal to 50.0% in the MYRROR report^16^. We used mean 2.11 aflibercept injections during one-year period, similar or less than mean 2.6 or 4.2 injections in the two preceding researches^16,18^. Most of the intravitreal aflibercept were administered in the first 3 months, similar to the report from the MYRROR study^16^. None of our patients reported serious adverse events during aflibercept treatment, comparable or even better than the safety profiles in the MYRROR study^16^.

Human choroidal neovascular membranes were found consistently expressing VEGF-A, VEGF-B, VEGF –C, and placental growth factor^26^. Knockdown of genes of placental growth factor or VEGF-A can inhibit the growth of laser-induced choroidal neovascularization in mice^27,28^. VEGF-B gene overexpression promoted pathological retinal and choroidal neovascularization in mice^29^. VEGF-A, VEGF-B, and placental growth factor were proven to have synergistic effects on pathologic angiogenesis^30^. Bevacizumab is a VEGF-A antibody, which can simply inhibit VEGF-A. Aflibercept is a compound simulating VEGF receptor, capable of downregulating not only VEGF-A, but also VEGF-B and placental growth factor. Because of different characteristics of two anti-VEGF agents, aflibercept had higher binding affinity of VEGF than bevacizumab in cell-based bioassays^31,32^. Aflibercept displayed a more prolonged VEGF inhibition in comparison with bevacizumab on retinal pigment epithelium/choroid organ cultures^33^. Compared to bevacizumab, topical applications or subconjunctival injections of aflibercept could inhibit corneal neovascularization for longer time period in a rat model^34,35^.

In our study, both visual and anatomical changes did not have significant differences between use of aflibercept and bevacizumab for mCNV. Intravitreal injections of either two anti-VEGF agents were mostly administered in the first three months, and more than half of the patients requiring no more bevacizumab or aflibercept injections in the second half of the year. The findings were analogous to those described in the prior studies, in which mCNV can be managed with a limited number of injections in the early course of treatment^6,8,16^. But injection number of aflibercept was significantly fewer than that of bevacizumab. In other words, aflibercept had more prolonged effect of treatment for our patients with mCNV than bevacizumab. This finding was compatible to the results of prior *in vitro* assays and animal experiments, that is, longer duration of action in aflibercept than that in bevacizumab^33–35^. Less frequent injections of aflibercept were beneficial to the patients with mCNV in several points, including less pain, less financial burden of the treatment, and less time-consuming load. Besides, fewer injection numbers may cause smaller possibility of intraoperative or postoperative adverse effects, such as retinal detachment, retinal pigment epithelial tears, infectious endophthalmitis, and thromboembolic events^2^.

To our knowledge, there is no publication comparing clinical outcome of aflibercept and bevacizumab for patients with mCNV. There were some limitations in the study. This is a retrospective, non-randomized, and comparative study performed in one institution. We had low sample size and possibly homogenous patient population. Some bias may occur in the setting of this study. A prospective, randomized, multi-centered trial will be required to prove the efficacy between two anti-VEGF agents.

In conclusion, both aflibercept and bevacizumab can effectively treat choroidal neovascularization in high myopes during one-year period. There was no significant difference in the visual and anatomical outcomes between two anti-VEGF agents. No serious systemic or ocular adverse events were observed in both anti-VEGF agents during study period.
